# Optimizing the formulation of *Erwinia* bacteriophages for improved UV stability and adsorption on apple leaves

**DOI:** 10.1016/j.heliyon.2023.e22034

**Published:** 2023-11-08

**Authors:** Su Jin Jo, Sang Guen Kim, Jungkum Park, Young Min Lee, Sib Sankar Giri, Sung Bin Lee, Won Joon Jung, Mae Hyun Hwang, Jae Hong Park, Eunjung Roh, Se Chang Park

**Affiliations:** a Laboratory of Aquatic Biomedicine, College of Veterinary Medicine and Research Institute for Veterinary Science, Seoul National University, Seoul, 08826, Republic of Korea; b Department of Biological Sciences, Kyonggi University, Suwon, 16227, Republic of Korea; c Crop Protection Division, National Institute of Agriculture Sciences, Rural Development Administration, Wanju, 55365, Republic of Korea

## Abstract

Fire blight is a bacterial disease that affects plants of the *Rosaceae* family and causes significant economic losses worldwide. Although antibiotics have been used to control the disease, concerns about their environmental impact and the potential to promote antibiotic resistance have arisen. Bacteriophages are being investigated as an alternative to antibiotics; however, their efficacy can be affected by environmental stresses, such as UV radiation. In this study, we optimized the formulation of *Erwinia* phages to enhance their stability in the field, focusing on improving their UV stability and adsorption using adjuvants. Our results confirmed that 4.5 % polysorbate 80 and kaolin improve phage stability under UV stress, resulting in an 80 % increase in PFU value and improved UV protection efficacy. Adsorption assays also demonstrated that polysorbate 80 and kaolin improved the absorption efficiency, with phages detected in plant for up to two weeks. These findings demonstrate the effectiveness of the auxiliary formulation of *Erwinia* bacteriophages against environmental stress.

## 1 Introduction

*Erwinia amylovora* is a Gram-negative bacterium that causes fire blight, a highly infectious and destructive bacterial disease that affects plants of the rose family *Rosaceae* [[1], [2], [3], [4]]. This bacterium is highly contagious and can infect a wide range of plant species, including fruit trees such as apples, pears, quince, and crabapples, as well as ornamental plants such as roses, pyracantha, and cotoneasters [5,6]. *Erwinia amylovora* enters plants through natural openings or wounds, such as pruning cuts or insect damage [7,8]. The symptoms of fire blight usually appear in spring and early summer when the weather is warm and moist [9]. Once inside the plant, the bacterium colonizes the vascular system, where it multiplies rapidly and produces a toxin that causes the plant tissue to turn brown or black, giving it the appearance of being scorched by fire [10,11]. Since it causes significant economic losses, *E. amylovora* is considered a serious threat to fruit production worldwide; total eradication in the field is not possible amid global warming [12,13]. Thus, rapid prevention and cultural practices control the spread of the outbreaks [14].

The general protocol against fire blight comprises antibiotics, such as streptomycin, oxytetracycline, and oxolinic acid during the bloom period [15,16]. As antibiotics for fire blight are shared between human and veterinary medicine, a considerable amount of antibiotic spillover may accumulate in the environment and potentially harm beneficial microorganisms, insects, and other organisms in the ecosystem [[17], [18], [19]]. Bacteriophages (phages) have been suggested as potential biocontrol agents, especially for controlling plant pathogenic bacterial species such as *Erwinia* [[20], [21], [22]]. A number of *Erwinia* phages have been characterized, and commercial phages for fire blight are available worldwide [[23], [24], [25], [26]]. Phages can be isolated in nature, are highly specific, and only kill their target bacterial pathogens, unlike antibiotics that cause dysbiosis in the rhizosphere and phyllosphere [[27], [28], [29]]. Furthermore, the combined use of phages and antibiotics has been suggested to reduce the use of antibiotics while optimizing the antimicrobial effect [30,31].

It is essential to consider the potential environmental stresses to optimize the antimicrobial effect of phages that may deteriorate their efficacy [32,33]. These factors include thermal stress, ultraviolet (UV) radiation, pH, and microbial competition [[34], [35], [36]]. These factors may affect the infection mechanisms of phages and physically degrade virion particles [37]. Auxiliary formulations, such as polysorbate 80, skim milk, casecrete, aromatic amino acids have been suggested to protect against environmental stress [38,39]. Remarkable concerns regarding the use of phages in agriculture include protection against UV radiation and adsorption on the surface of plant materials [33,40,41]. Polysorbate 80 and kaolin are two widely used agricultural surfactants [42,43]. Polysorbate 80, an emulsifier and dispersant, reduces bacterial attachment and inhibit biofilm formation on the plant surface [44]. Kaolin, a natural mineral clay, is used in agriculture to form a protective barrier that helps ward off insect-pests and reduce UV stress [45]. It is adsorbed on the plant surface, generating a white film that distracts insects and spreads pathogens. Moreover, the film deflects ultraviolet rays and reduces the heat stress of plants [46,47]. Optimized formulations containing these chemicals are expected to improve the distribution and prolongation of these biocontrol agents on plant surfaces, thus enhancing their efficacy in controlling fire blight under challenging environmental conditions.

In this study, we focused on protecting phages from pivotal stress factors in the agricultural field, such as ultraviolet radiation and the adsorption of phages on the plant surface. The UV protection and adsorption effects of polysorbate 80, kaolin, and their combinations as auxiliary agents were also examined.

## 2 Results

### 2.1 Phage morphology

**Fig. 1:**
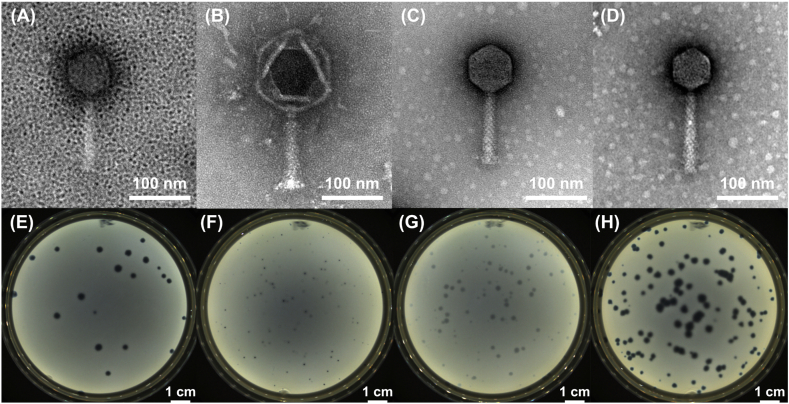
**Transmission electron microscopy image (A**–**D) and plaque morphology (*E***–**H) of *Erwinia amylovora* phages used in this study.** (A) pEa_SNUABM_27, (B) pEa_SNUABM_47, (C) Fifi318, (D) Fifi451, (E) pEa_SNUABM_27, (F) pEa_SNUABM_47, (G) Fifi318, and (H) Fifi451.

The morphology of the phages pEa_SNUABM_27 (φ27), pEa_SNUABM_47 (φ47), Fifi318 (φ318), and Fifi451 (φ451) was determined through transmission electron microscopy. The phages were found to have capsids with diameters of 68.5 ± 2.76, 127.74 ± 6.58, 74.35 ± 2.97, and 62.28 ± 1.23 nm, respectively (Fig. 1 (A) ∼ (D)). They were observed to have contractile tails of approximately 115.1 ± 2, 196.32 ± 11.45, 108.97 ± 5.49, and 127.67 ± 3.1 nm in length, respectively. Based on their morphology, the phages were assigned to the *Myoviridae* family. The phages were observed to have plaque sizes of 0.34, 0.09, 0.19, and 0.32 cm, respectively (Fig. 1 (E) ∼ (H)).

### 2.2 UV protection by polysorbate 80

**Fig. 2:**
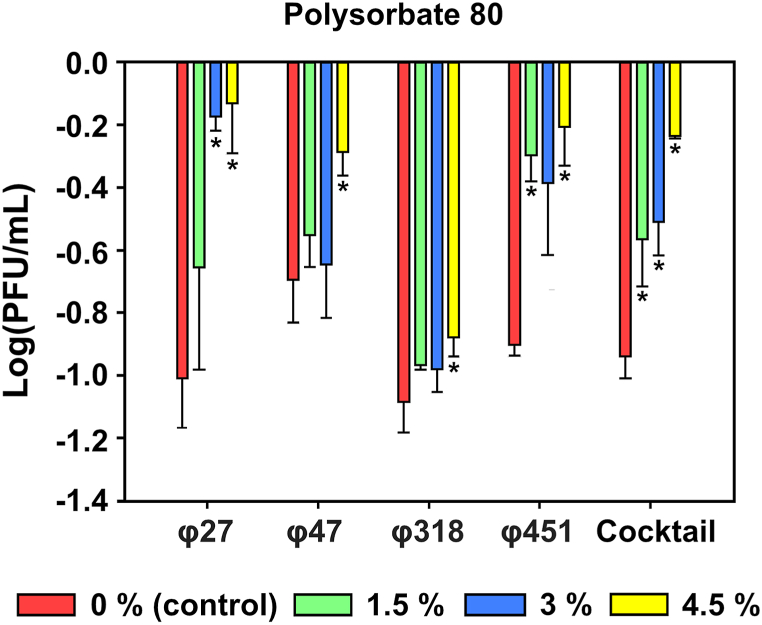
**UV protection effect of polysorbate 80 on the representative *Erwinia amylovora* phages.** The phage titer was determined using the indicator host strain (TS 3128). Statistical analysis was performed using One-way ANOVA with Dunnett's test to determine significant differences (n = 3).

Plaque Forming Unit (PFU) titers were measured to confirm the effect of polysorbate 80 on phage protection against UV radiation. After treating the phages with various concentrations of polysorbate 80, they were exposed to UV light for 6 h (Fig. 2). In the control group, φ27 showed one logPFU/mL reduction, whereas phages treated with 1.5-, 3-, and 4.5 % polysorbate 80 showed reductions of 0.65-, 0.17-, and 0.12 logPFU/mL, respectively. For φ47, the control showed a reduction of 0.69 logPFU/mL, and reductions of 0.55-, 0.64-, and 0.28 logPFU/mL were observed in the treatment groups with increasing polysorbate 80 concentrations (1.5-, 3-, and 4.5 %, respectively). φ318 was reduced by 1.08 logPFU/mL in the control group, whereas phages treated with 1.5-, 3-, and 4.5 % polysorbate 80 had modest reductions of 0.96-, 0.97-, and 0.87 logPFU/mL. φ451 showed a reduction of 0.77 logPFU/mL in the control group, and reductions of 0.29-, 0.38-, and 0.20 logPFU/mL were observed in the treatment groups (1.5-, 3-, and 4.5 %, respectively). The phage cocktail showed a reduction of 0.93 logPFU/mL in the control, and reductions of 0.56-, 0.50-, and 0.23 logPFU/mL were observed in the treatment groups (1.5-, 3-, and 4.5 %, respectively). Adding polysorbate 80 protected the phage against UV exposure, and the most effective concentration was 4.5 % for all tested representative *Erwinia amylovora* phages.

### 2.3 UV protection by kaolin

**Fig. 3:**
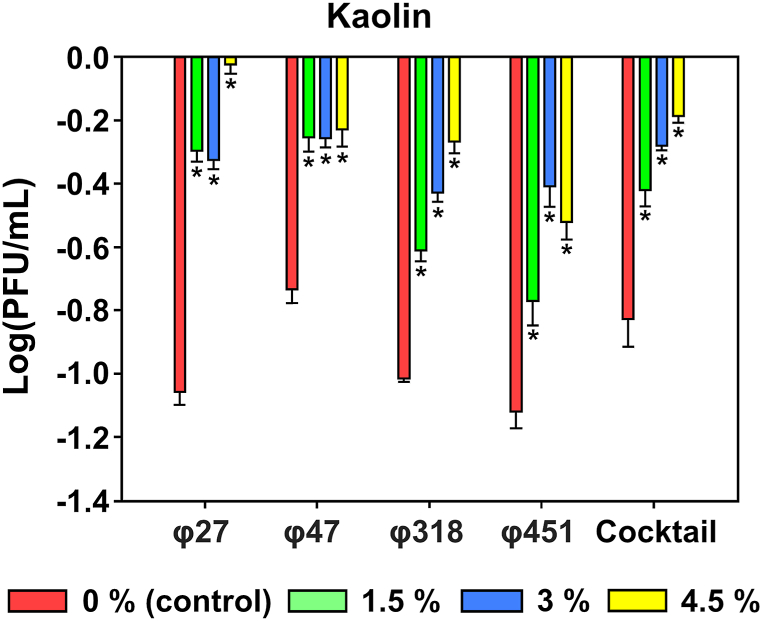
**UV protection effect of kaolin on the representative *Erwinia amylovora* phages.** The phage titer was determined using the indicator host strain (TS 3128). Statistical analysis was performed using One-way ANOVA with Dunnett's test to identify significant differences (n = 3).

To test the effect of UV on phages and the efficacy of the auxiliary agents, we added kaolin to the phage at concentrations of 0 (control), 1.5-, 3-, and 4.5 %. Six hours of treatment with kaolin showed significant effects on all phages (Fig. 3). For φ27, the control decreased by 1.05 logPFU/mL, while adding 1.5-, 3-, and 4.5 % kaolin resulted in reductions of 0.29-, 0.32-, and 0.02 logPFU/mL, respectively. The titer of φ47 decreased by 0.73 logPFU/mL in the untreated control, and by 0.25-, 0.25-, and 0.22 logPFU/mL after treatment with 1.5-, 3-, and 4.5 % kaolin, respectively. φ318 showed a 1.01 logPFU/mL decrease in the control and 0.6-, 0.42-, and 0.26 logPFU/mL after adding 1.5-, 3-, and 4.5 % kaolin, respectively. Concentration of φ451 decreased 1.11 logPFU/mL in control group, and 0.76-, 0.40-, and 0.51 logPFU/mL with 1.5-, 3-, and 4.5 % kaolin treatment, respectively. In the four phages cocktail, the control decreased by 0.82 logPFU/mL, while 0.41-, 0.27-, and 0.18 logPFU/mL reductions were observed in the 1.5-, 3-, and 4.5 % kaolin-treated groups. After conducting tests, it was found that an optimal concentration of 4.5 % of kaolin was effective in providing protection against UV exposure for representative *Erwinia amylovora* phages tested.

### 2.4 UV protection by a combination of polysorbate 80/kaolin

**Fig. 4:**
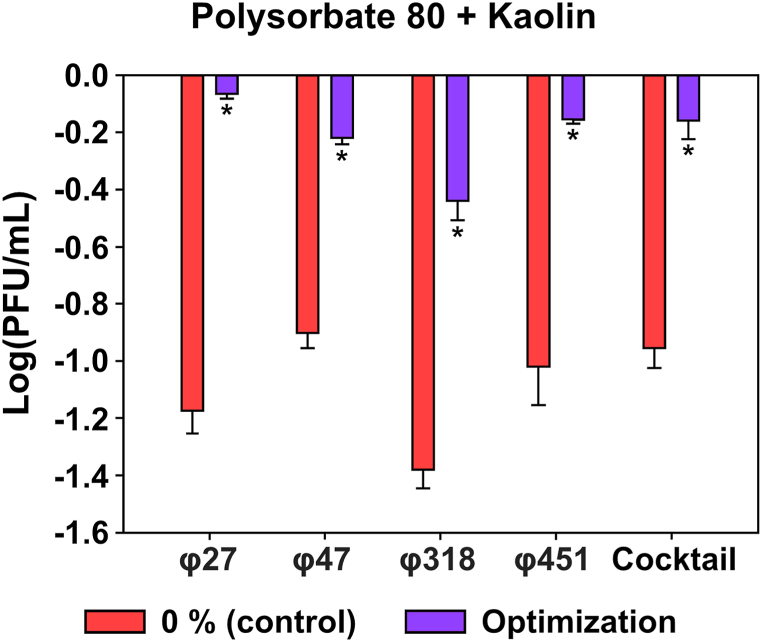
**UV protection effect of the formulation on the representative *Erwinia amylovora* phages.** The optimal concentration, 4.5 % w/w, was used. Bacteriophages were incubated with the combination of polysorbate 80 and kaolin under UV radiation for 6 h. The ability of the phages to form plaques on host lawns (TS 3128) was assessed, and statistical significance was determined using One-way ANOVA with Dunnett's test (n = 3).

After conducting two UV stability tests, we confirmed that the best protective effect was achieved when polysorbate 80 and kaolin were mixed with the phages at a concentration of 4.5 %. We then tested the UV protection effect of the optimized concentrations of the two substances for 6 h (Fig. 4). The results showed that phages φ27, φ47, φ318, φ451, and the phage cocktail control decreased by 1.17-, 0.90-, 1.37-, 1.01-, and 0.95 logPFU/mL, respectively, whereas the treatment group decreased by 0.06-, 0.21-, 0.43-, 0.15-, and 0.15 logPFU/mL, respectively. All phages exhibited significant (*p* < 0.05) results, indicating that the combined formulation effectively protected phages from UV stress.

### 2.5 Auxiliary effect of the combined formulation in plant tissue

**Fig. 5:**
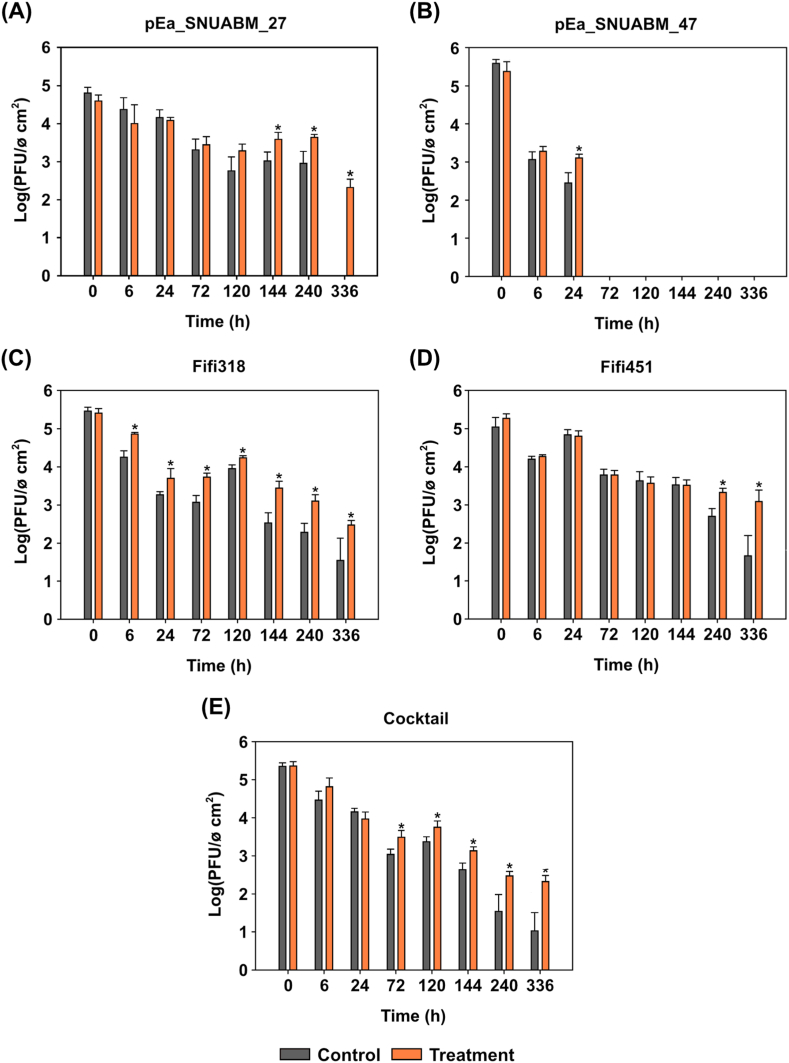
**Adsorption efficacy of the formulation on the leaf surface.** Random samples of the plant tissue over a two-week period were assessed. The statistical significance was examined using One-way ANOVA with Dunnett's test (n = 4).

We then evaluated the effectiveness of the formulation of the two auxiliary agents for foliar adsorption. We applied phages coated with 4.5 % polysorbate 80 and kaolin to leaves and tested them for two weeks. Most phages applied with auxiliary agents were detected for over two weeks (Fig. 5). Samples mixed with phage φ27 and the auxiliary agents showed significant differences (*p* = 0.033) from day seven onwards, with a phage concentration difference of 2.3 logPFU/mL from the control group by 336 h (Day 14). Although the phage φ47 was not adsorbed for a long time, phages containing auxiliary agents showed a significant difference (*p* = 0.003) of 0.6 logPFU/mL up to 24 h. Phage φ318 showed no difference at zero hours but showed significant differences (*p* = 0.010) in phage-containing auxiliary agents for up to 2 weeks, and the treated group was observed to be more effective. By 336 h (Day 14), a 2.4 logPFU/mL phage concentration was detected, while the control was 1.5 logPFU/mL. Phage φ451 showed a rapid decrease in PFU after ten days in the control group; however, a significant difference (*p* = 0.008 and *p* = 0.037, respectively) in PFU was observed in the formulation treatment group after 240 h (Day 10) to 336 h (Day 14). The phage cocktail showed a significant difference (*p* = 0.025) from 72 h (Day 3), followed by a growing difference from 240 h (Day 10) onwards. In the formulation treated group, phages were often detected even on the 21st day (data not shown). However, most untreated phages (controls) showed a marked decrease from Day ten onwards. Our findings suggest that auxiliary agents are effective in facilitating foliar phage adsorption.

## 3 Discussion

The effectiveness of phages as biocontrol agents for fire blight has been established in previous studies [22,[48], [49], [50]]; however, various stress factors limit their efficacy in real-world environments [33]. These stress factors include the host immune system, microbial competitors, and exposure to UV radiation [32]. This study aimed to develop an auxiliary agent to prolong the survival of *Erwinia* phage to address the UV radiation and adsorption issues.

A number of *Erwinia* phages have been reported so far. Myovirus morphology phages having rigid contractile tail fiber are the majority, while phages having podovirus and siphovirus morphology have been rarely reported [[51], [52], [53]]. The present study used previously isolated phages as representatives of myophages, *Lossenervirus* for pEa_SNUABM_27 [30], and Fifi451 [54]; *Eneladusvirus* for pEa_SNUABM_47 [23]; unknown for Fifi318. Phages are reportedly susceptible to UV radiation [34,55]. However, most studies assessing phage UV resistance used UV-C at a wavelength of 254 nm, which is predominantly absorbed by the ozone layer [39,56,57]. UV-C radiation is typically absorbed by the Earth's atmosphere and does not reach the Earth's surface [58]. Most solar UV radiation reaching the Earth's surface comprises longer-wavelength UV-A and UV-B rays, which have minimal effects on DNA damage [59]. Likewise, the previous study that employed UV-C for the UV protection assay, the protection efficacy of the auxiliary agents could not be appropriately appreciated [30]. To simulate the actual damage phages might receive from sunlight, we employed a solar simulator capable of emitting light in the 270–400 nm range, encompassing all wavelengths from UV-A, UV-B, and UV-C [60]. In contrast to a previous study that used UV-C and showed a weak protection effect of polysorbate 80 [39], our study demonstrated that it could exert much stronger effect. Only 0.5 % of phages sustained the infectivity after a short period of time (5 min) by UV-C irradiation, however, the protection effect of polysorbate can be improved showing up to 78 % of survival rate of phages even after 6 h of 1 SUN UV irradiation (Fig. 2). Thus, using a solar simulator to assess phage stability is recommended as a suitable method for evaluating the efficacy of phages intended for use in agriculture. Our findings suggest that phages might be more stable under UV irradiation with the formulation proposed in the present study.

In addition to the UV protection effect demonstrated by auxiliary agents, polysorbate 80 is generally recognized as a safe (GRAS) nonionic surfactant with low toxicity and cost, making it suitable for use in agriculture [61]. Moreover, it can destroy the structure of specific bacterial cell membrane proteins and lipid components, making it a promising auxiliary agent for phage-based biocontrol [62].

Although kaolin is known as a UV-reflective film, a few studies have investigated its application to phages [63]. When applied to crops, kaolin forms a white film on the leaves, reducing the amount of light that reaches the plant surface by reflecting it [64]. Notably, the UV protective effect of kaolin was observed for the representative phages by preventing the loss of infectivity (Fig. 3). Our findings suggest that kaolin is repellent in preventing pests and can reduce UV damage to phages. Hence, our study offers new insights into the potential application of kaolin as an auxiliary phage-based biocontrol agent in agricultural applications.

One of the critical factors that should be considered for applying phages as biocontrol agents is their fate on targeted environment [33,37,48,65]. The plant surface is characterized by its anti-adhesive nature, which makes it difficult for phages to adhere and to penetrate the plant tissues [66]. To address this issue, we examined the optimized conditions of the polysorbate and kaolin combination for their efficacy in prolonging the fate of phages for two weeks. The results showed that combining these two auxiliary formulations considerably improved the persistence of phages on the plant surface (Fig. 4), as evidenced by the higher PFU values compared to the control, which showed rapid eradication within a few days, as previously reported (Fig. 5) [39,40]. We performed this experiment on plant tissue. Therefore, the secondary effect of phage uptake on physiological changes *in planta* should be investigated in future studies.

Administering anti-fire blight agents are typically recommended once every 1–2 weeks [67]. Our results suggest that the formulation developed in this study has the potential to be applied as an auxiliary agent for the agricultural therapeutic use of phages. However, further investigations, such as repeated exposure to UV stress, testing with actual plants and environments, and testing other combinations with other appropriate materials, should be conducted in future studies to address potential issues.

## 4 Materials and methods

### 4.1 Microorganisms and culture conditions

The sole reference strain allowed for research in South Korea, *Erwinia amylovora* TS3128 was used in the study. Previously reported phages, pEa_SNUABM_27 and pEa_SNUABM_47, were isolated from the river near diseased orchard and used in further assays [23,30]. Phage Fifi318 and Fifi451 were kindly provided by Dr. Eunjung Roh from the Rural Development Administration of Korea. The microorganisms were cultured in tryptic soy agar (TSA) and TSB supplemented with 0.4 % agar as a top agar for phage cultivation at 27 °C for 18–24 h [68]. The overnight (∼18 h) grew bacterial cell culture was used for lawn cells. And serial dilution was conducted with SM buffer (50 mM Tris [pH 7.5], 100 mM NaCl, and 10 mM MgSO_4_).

### 4.2 Phage propagation and purification

After amplifying the phages using the double agar overlay plaque assay described in a previous publication [69], the top agar layer containing the phages was mixed with an SM buffer was incubated at 27 °C for 24 h. The resulting phage solution was then centrifuged at 12,000×*g* for 10 min, and the supernatant was mixed with 10 % (w/v) polyethylene glycol/0.5 M NaCl (final concentration) to precipitate the phages. Cesium chloride (CsCl) density gradient centrifugation was used to purify the phage particles [70]. The phage samples were ultracentrifuged for 3 h at 50,000×*g* using a Type 70 Ti fixed-angle titanium rotor (Beckman, Brea, CA, USA), and the resulting phage bands were collected. The purified samples (>10^10^ PFU/mL) were then dialyzed using a 7000 MWCO Slide-A-Lyzer® Dialysis Cassette (Thermo Scientific, Waltham, MA, USA). The dialyzed samples were filtered using a 0.22 μm filter (Millipore) and stored at 4 °C.

### 4.3 Transmission electron microscopy

The purified phage suspensions were combined with an equal amount of 2 % uranyl acetate (10 μL) to prepare for transmission electron microscopy analysis [71]. After 1 min, the excess sample was eliminated, and the grids were washed with distilled water. The grids were air-dried for an hour before morphological analysis of the phages was performed using a Talos L120C transmission electron microscope (FEI, Hillsboro, OR, USA) operating at 120 kV. The size of the isolated virions and plaque was measured, and the average phage and plaque size was calculated.

### 4.4 UV protection assay

Bacteriophages were prepared at a density of 4 × 10^8^ PFU/mL suspended in SM buffer in 96 well plates. The phage cocktail consisted of φ27, φ47, φ318, and φ451 mixed at the same proportions (1:1:1:1). The phages were mixed with the double the concentration of auxiliary agents, kaolin (Sigma-Aldrich) or polysorbate 80 (Sigma-Aldrich), and mixed 1:1 in a 96-well plate to make a final concentration of 2 × 10^8^ PFU/mL and auxiliary agents at 0 % (control), 1.5 %, 3 %, and 4.5 % (w/v). For prevention of drying and contamination, the 96-well plate was covered with a quartz square plate (1 mm depth) which is known to not block UV rays. The plate was half submerged in water and kept at constant temperature at 25 °C with continuous circulation of water. The phage sample mixed with the auxiliary agent was exposed to UV light for 6 h using a solar simulator (Newport 94,023 A) equipped with a 450 W xenon lamp and an intensity of 1 SUN (100 mW/cm^2^). Phage concentration in the sample concentrations was evaluated in triplicate using a double agar overlay plaque assay to observe a decline in phage. *Erwinia amylovora* TS3128 was used as an indicator host strain for the plaque assay. For the optimization of the formulation for the UV protection effect, we used the best combination of the minimum decline in phage concentration obtained in the UV protection assay with the mixing of auxiliary agents (kaolin and polysorbate 80) from the UV protection assay were employed. A mixed sample with a final concentration of 4.5 % of both auxiliary agents was distributed in 96-well plates and exposed to UV light for 6 h. Phage concentration in the sample was evaluated in triplicate using the plaque assay using *E. amylovora* TS3128 used as an indicator host strain.

### 4.5 Adsorption in plants tissues

To evaluate the effects of phages and phage cocktails containing auxiliary agents, plant tissues (leaves of Fuji apple) were sterilized with sodium hypochlorite, washed with distilled water, and dried at the biosafety cabinet. After conducting the UV stability test, the best concentrations of kaolin and polysorbate 80 was mixed with 10 mL of an SM buffer solution. Tissues were soaked in this solution for 3 min and then dried at 25 °C for 30 min. The dried tissues were stored at 25 °C and 50–60 % humidity for the test. Samples of 0.09 ø cm^2^ were obtained at 0 (control), 6, 24, 72, 120, 168, 240, and 336 h post-treatment using a 6 mm biopsy punch (n = 4). The sample was then incubated in 1 mL of SM buffer for 30 min with shaking and vortexed for 30 s. *E. amylovora* TS3128 was used as an indicator host strain to enumerate the PFUs in the tissues.

### 4.6 Statistical analysis

Each experimental set of data of UV protection assay, and adsorption in plants tissues was statistically analyzed with one-way analysis of variance (ANOVA) and the Dunnett's test using SigmaPlot software version 12.5 (Systat Software, San Jose, CA, USA).

## Data availability statement

Data will be made available on request.

## Additional information

No additional information is available for this paper.

## CRediT authorship contribution statement

**Su Jin Jo:** Conceptualization, Data curation, Formal analysis, Investigation, Methodology, Writing – original draft, Writing – review & editing. **Sang Guen Kim:** Conceptualization, Data curation, Formal analysis, Investigation, Methodology, Project administration, Software, Supervision, Validation, Visualization, Writing – original draft, Writing – review & editing. **Jungkum Park:** Formal analysis, Investigation, Methodology, Resources, Validation. **Young Min Lee:** Data curation, Formal analysis, Methodology, Resources, Writing – review & editing. **Sib Sankar Giri:** Conceptualization, Data curation, Investigation, Validation, Writing – review & editing. **Sung Bin Lee:** Resources, Validation, Writing – review & editing. **Won Joon Jung:** Investigation, Resources, Writing – review & editing. **Mae Hyun Hwang:** Investigation, Methodology, Resources, Writing – review & editing. **Jae Hong Park:** Investigation, Resources, Validation, Writing – review & editing. **Eunjung Roh:** Conceptualization, Data curation, Investigation, Validation, Writing – review & editing. **Se Chang Park:** Conceptualization, Data curation, Formal analysis, Funding acquisition, Supervision, Writing – review & editing.

## Declaration of competing interest

The authors declare that they have no known competing financial interests or personal relationships that could have appeared to influence the work reported in this paper.
